# Postmenopausal circulating levels of 2- and 16*α*-hydroxyestrone and risk of endometrial cancer

**DOI:** 10.1038/bjc.2011.381

**Published:** 2011-09-27

**Authors:** A Zeleniuch-Jacquotte, R E Shore, Y Afanasyeva, A Lukanova, S Sieri, K L Koenig, A Idahl, V Krogh, M Liu, N Ohlson, P Muti, A A Arslan, P Lenner, F Berrino, G Hallmans, P Toniolo, E Lundin

**Affiliations:** 1 Department of Environmental Medicine, New York University School of Medicine, 650 First Avenue, New York, NY 10016, USA; 2 New York University Cancer Institute, New York University School of Medicine, 530 First Avenue, New York, NY 10016, USA; 3 Radiation Effects Research Foundation, 5-2 Hijiyama Park, Minami-ku, Hiroshima, 732-0815, Japan; 4 Division of Cancer Epidemiology, German Cancer Research Center (DKFZ), Im Neuenheimer Feld 280, D-69120, Heidelberg, Germany; 5 Department of Obstetrics and Gynecology, New York University School of Medicine, 550 First Avenue, New York, NY 10016, USA; 6 Department of Preventive and Predictive Medicine, Nutritional Epidemiology Unit, Fondazione IRCCS Istituto Nationale dei Tumori, Via Venezian, 1, 20133, Milan, Italy; 7 Department of Clinical Science, Obstetrics and Gynecology, Umeå University Hospital, S-901 85, Umeå, Sweden; 8 Department of Medical Biosciences/Pathology, Umeå University, SE-901 87, Umeå, Sweden; 9 Istituto Nazionale Tumori Regina Elena, Via E. Chianesi 53, 00144, Rome, Italy; 10 Department of Radiation Sciences, Oncology, Umeå University, S-90185, Umeå, Sweden; 11 Department of Preventive and Predictive Medicine, Epidemiology Unit, Fondazione IRCCS Istituto Nazionale dei Tumori, Via Venezian, 1, 20133, Milan, Italy; 12 Department of Public Health and Clinical Medicine/Nutritional Research, Umeå University, SE-901 87, Umeå, Sweden; 13 Unit of Cancer Epidemiology, Institute of Social and Preventive Medicine, Centre Hospitalier Universitaire Vaudois, Biopôle 1, 2 Route de la Corniche, CH-1066 Epalinges, Switzerland

**Keywords:** endometrial cancer, nested case–control study, oestrogen metabolites, 2-hydroxyestrone, 16*α*-hydroxyestrone, post-menopause

## Abstract

**Background:**

It has been suggested that the relative importance of oestrogen-metabolising pathways may affect the risk of oestrogen-dependent tumours including endometrial cancer. One hypothesis is that the 2-hydroxy pathway is protective, whereas the 16*α*-hydroxy pathway is harmful.

**Methods:**

We conducted a case–control study nested within three prospective cohorts to assess whether the circulating 2-hydroxyestrone : 16*α*-hydroxyestrone (2-OHE1 : 16*α*-OHE1) ratio is inversely associated with endometrial cancer risk in postmenopausal women. A total of 179 cases and 336 controls, matching cases on cohort, age and date of blood donation, were included. Levels of 2-OHE1 and 16*α*-OHE1 were measured using a monoclonal antibody-based enzyme assay.

**Results:**

Endometrial cancer risk increased with increasing levels of both metabolites, with odds ratios in the top tertiles of 2.4 (95% CI=1.3, 4.6; *P*_trend_=0.007) for 2-OHE1 and 1.9 (95% CI=1.1, 3.5; *P*_trend_=0.03) for 16*α*-OHE1 in analyses adjusting for endometrial cancer risk factors. These associations were attenuated and no longer statistically significant after further adjustment for oestrone or oestradiol levels. No significant association was observed for the 2-OHE1 : 16*α*-OHE1 ratio.

**Conclusion:**

Our results do not support the hypothesis that greater metabolism of oestrogen via the 2-OH pathway, relative to the 16*α*-OH pathway, protects against endometrial cancer.

The role of oestrogens in the development of endometrial cancer is well documented by both experimental and epidemiologic studies (Key and Pike, 1988; Akhmedkhanov *et al*, 2001). High oestrogen, relative to progesterone/progestin, exposure leads to endometrial hyperplasia, followed by atypical hyperplasia, the precursor of most endometrial cancers (Sherman, 2000). In addition to exogenous oestrogens (Grady *et al*, 1995), circulating levels of endogenous oestrogens in postmenopausal women have been shown to be positively associated with risk of endometrial cancer in prospective studies (Zeleniuch-Jacquotte *et al*, 2001; Lukanova *et al*, 2004; Allen *et al*, 2008). Oestrogens are metabolised through various pathways, and it has been suggested that the relative importance of these pathways within an individual may affect the risk of oestrogen-dependent tumours, such as breast and endometrial cancers (Fishman *et al*, 1984; Zhu and Conney, 1998). One specific hypothesis is that the 2-hydroxyestrogen metabolite pathway is protective, whereas the 16*α*-hydroxyestrogen pathway is harmful (Schneider *et al*, 1982; Fishman *et al*, 1984; Sepkovic and Bradlow, 2009). The basis for this hypothesis is that 2-hydroxyestrogens bind to oestrogen receptors but do not activate transcription, resulting in no or limited proliferative effects, whereas 16*α*-hydroxyestrogens have prolonged oestrogenic effects including stimulation of cell proliferation (Sepkovic and Bradlow, 2009).

A number of prospective studies have examined the effects of 2-hydroxyestrone (2-OHE1), 16*α*-hydroxyestrone (16*α*-OHE1) and their ratio in relation to risk of breast cancer with inconsistent results (Meilahn *et al*, 1998; Muti *et al*, 2000; Cauley *et al*, 2003; Wellejus *et al*, 2005; Modugno *et al*, 2006; Eliassen *et al*, 2008; Arslan *et al*, 2009). No study, though, has been conducted in relation to endometrial cancer risk. We conducted a case–control study nested within three prospective cohorts to test the hypothesis that the circulating 2-OHE1 : 16*α*-OHE1 ratio, an index of the relative importance of the 2-OH and 16*α*-OH oestrogen pathways, is inversely associated with risk of endometrial cancer in postmenopausal women. We previously reported positive associations between postmenopausal circulating oestrogens and androgens and risk of endometrial cancer in a subset of the subjects included in this study (Lukanova *et al*, 2004).

## Materials And Methods

### Study population and biological sample collection

The case–control study was nested within three cohorts: the Northern Sweden Disease and Health Study (NSHDS) in Umeå, Sweden (Hulten *et al*, 2001), the New York University Women's Health Study (NYUWHS) in New York City, NY, USA (Toniolo *et al*, 1995; Zeleniuch-Jacquotte *et al*, 2001) and the ORDET cohort in Milan, Italy (Muti *et al*, 2000; Sieri *et al*, 2009). The recruitment setting, eligibility criteria and case ascertainment methods for each cohort are described in Table 1. As described in Lukanova *et al* (2004), data on known risk factors for endometrial cancer were obtained at enrolment through interviews by nurses for ORDET and by a combination of self-administered baseline and follow-up questionnaires for the NSHDS and the NYUWHS. Height and weight were self-reported for the NSHDS and the NYUWHS and measured by nurses for ORDET at enrolment. Venous blood was collected at enrolment (plasma for the NSHDS, serum for the NYUWHS and both plasma and serum for ORDET). All specimens were stored at −80°C by the respective cohorts. The Institutional Review Boards of New York University School of Medicine and of the Istituto Nazionale dei Tumori in Milan and the Regional Ethical Committee of the University of Umeå, Sweden, reviewed and approved this study. Informed consent was obtained from all participants at enrolment.

Menopausal status was determined at enrolment for the NYUWHS and ORDET participants. Women were classified as postmenopausal if they had not had menstrual periods for at least 6 months (NYUWHS) or 12 months (ORDET). In the NYUWHS, women who had had a total oophorectomy and women who had had a hysterectomy without total oophorectomy and were 52 years old or older were also classified as postmenopausal. In ORDET, women who had had a bilateral oophorectomy were not eligible to enter the cohort. For NSHDS cases and controls, menopausal status at enrolment was determined retrospectively based on reported age at menopause on baseline or case–control questionnaires. In addition, FSH measurements were used for all women between the ages of 46 and 57 years. Women were classified as postmenopausal if they had FSH levels >20 IU l^−1^ and their questionnaire data were consistent with postmenopausal status. Finally, women taking hormone replacement therapy (HRT) in the three (ORDET), or six (NSHDS, NYUWHS), months before enrolment were not included.

### Case ascertainment and control selection

Case subjects were women who were postmenopausal at enrolment and diagnosed with invasive endometrial cancer (ICD-O codes 8010, 8140, 8210, 8260, 8310, 8323, 8380, 8382, 8441, 8460, 8461, 8480, 8481, 8560 and 8570) after enrolment and before 1 January 2007 for the NSHDS, 1 July 2003 for the NYUWHS and 1 January 2004 for ORDET. Cases who had been diagnosed with another cancer (except non-melanoma skin cancer) before endometrial cancer were not eligible.

For each case, two controls were selected at random from the appropriate risk set, which consisted of all women with an intact uterus and free of cancer at the date of diagnosis of the case and matching the case on cohort, menopausal status at enrolment, age (±6 months) at, and date (±3 months) of, blood donation.

### Laboratory analyses

All oestrogen metabolite assays were conducted in the same laboratory, Immuna Care Corporation, (Tampa, FL, USA), using monoclonal antibody-based enzyme assays (ESTRAMET 2/16, Immuna Care Corporation). The enzyme immunoassays (EIAs) were developed for urinary samples (Klug *et al*, 1994; Falk *et al*, 2000) and validated against gas chromatography–mass spectrometry (GC–MS) (Falk *et al*, 2000). They were validated for serum by conducting the assays on pooled human serum samples to which known amounts of 2-OHE1 and 16*α*-OHE1 had been added (Klug, personal communication). Serum samples were used for the NYUWHS and ORDET, and EDTA-plasma samples for the NSHDS. Assay variability was assessed by including 10% blinded quality control samples (samples from a pool for the NSHDS and duplicates for the NYUWHS). For serum, the within- and between-batch coefficients of variation were 4% and 6% for 2-OHE1 and 1% and 2% for 16*α*-OHE1, respectively. For EDTA-plasma, the within- and between-batch coefficients of variation were 2% and 2% for 2-OHE1 and 1% and 4% for 16*α*-OHE1, respectively.

As described previously (Lukanova *et al*, 2004), oestrone and oestradiol were measured by a radioimmunoassay following organic extraction and celite chromatography for 129 (76%) of the NYUWHS samples. For all other samples, oestrone was measured by double antibody RIA and oestradiol by ultrasensitive double antibody RIA with reagents from Diagnostic Systems Laboratories (Webster, TX, USA).

### Statistical methods

The intra-class correlation coefficient (Donner, 1986) was used to assess the temporal reliability of the oestrogen metabolites and their ratio in a pilot study where oestrogen metabolites were measured in three serum samples collected at approximately yearly intervals in 30 NYUWHS participants postmenopausal at blood donation and free of cancer at latest follow-up.

Because serum was used for the NYUWHS and the ORDET cohorts and EDTA plasma for the NSHDS, a substudy was conducted to compare levels of oestrogen metabolites in serum with levels in EDTA-plasma samples collected at the same visit from 17 NSHDS participants. We found that plasma samples led to much higher values than serum samples: for 2-OHE1 the median was 526 pg ml^−1^ using plasma and 183 pg ml^−1^ using serum, and for 16*α*-OHE1 the median was 618 pg ml^−1^ using plasma and 518 pg ml^−1^ using serum. The median 2-OHE1 : 16*α*-OHE1 ratio was 1.06 using plasma samples and 0.43 using serum samples. Although the absolute values were quite different, the Pearson correlation coefficients were high between plasma and serum levels (0.96 for 2-OHE1, 0.99 for 16*α*-OHE1 and 0.81 for the 2-OHE1 : 16*α*-OHE1 ratio). Further analyses showed that serum levels (on the natural logarithmic scale) of metabolites could be accurately predicted from plasma levels (also on the logarithmic scale) using a linear regression model (*R*^2^=0.85 for 2-OHE1 and 0.99 for 16*α*-OHE1). The observed serum 2-OHE1 : 16*α*-OHE1 ratio was slightly more strongly correlated (*r*=0.86) with the predicted serum ratio (calculated as the ratio of the predicted serum 2-OHE1 and 16*α*-OHE1 levels) than with the plasma ratio (*r*= 0.81). Therefore, and in order to facilitate the combination of the data from the three cohorts as well as the interpretation of the results, we used predicted serum values for the NSHDS participants in all analyses. To predict the serum levels of 2-OHE1 and 16*α*-OHE1 from the plasma levels for the NSHDS samples, we created 10 imputed data sets using a multiple imputation procedure with the linear regression models fitted in the substudy. We then calculated the serum 2-OHE1 : 16*α*-OHE1 ratio using the imputed serum levels of 2-OHE1 and 16*α*-OHE1. The SAS PROC MI and MIANALYZE in SAS 9.2 were used (SAS Institute, Inc., Cary, NC, USA).

The conditional logistic regression model was used to take into account the matched design. The analysis was conducted after classifying participants according to cohort-specific metabolite tertiles using the distribution of cases and controls combined. The lowest tertile was used as reference. Because analyses were conducted using 10 imputed data sets, the means of the odds ratio estimates are reported. The within-imputation variance and between-imputation variances were calculated and combined to yield the total variance using Rubin's method (Rubin, 1987). The 95% confidence intervals and *P*-values were then calculated using the *t*-distribution (SAS PROC MI and MIANALYZE). In addition to analyses by tertiles, analyses were conducted with the oestrogen metabolites and their ratio on the continuous (log transformed) scale. The log_2_ transformation, which leads to an odds ratio for a doubling in level, was used.

The following known endometrial cancer risk factors were considered as potential confounders: age at menarche, parity, OC use, HRT use, body mass index (BMI) and smoking. Analyses adjusting simultaneously for all these variables are presented in addition to unadjusted analyses. Analyses stratified by age at sampling (<60 and ⩾60 years), age at diagnosis (<65 and ⩾65 years), lag time between blood donation and diagnosis (<7 and ⩾7 years) and BMI (<25 and ⩾25 kg m^−2^) were also conducted. Interaction tests were conducted to assess whether the associations of the oestrogen metabolites or their ratio with endometrial cancer risk varied according to strata of these factors. We also assessed whether the oestrogen metabolite–endometrial cancer risk associations differed by cohort.

## Results

The ICC measuring the temporal reliability over a 2-year period was 0.62 (95% CI=0.42, 0.78) for 2-OHE1, 0.95 (95% CI=0.91, 0.97) for 16*α*-OHE1 and 0.69 (95% CI=0.52, 0.82) for their ratio. These results were quite similar to those observed by Eliassen *et al* who reported intra-class correlation coefficients of 0.63 for 2-OHE1, 0.80 for 16*α*-OHE1 and 0.73 for their ratio, indicating that a single measurement can be used as a marker of average exposure to these metabolites in epidemiologic studies (Eliassen *et al*, 2008).

A total of 179 cases and 336 controls were included (89 cases and 164 controls from the NSHDS, 75 cases and 143 controls from the NYUWHS and 15 cases and 29 controls from ORDET). Table 2 reports on the case and control subject characteristics. The median age at blood donation was 59.9 years and at diagnosis 66.5 years. The vast majority of participants (94% of cases and 97% of controls) were Caucasians. Associations with known risk factors for endometrial cancer were in the expected directions. Compared with controls, cases had younger age at menarche and older age at menopause, and were heavier. Cases were more likely than controls to have used HRT but less likely to have used oral contraceptives and to have ever smoked. Levels of both oestrogen metabolites were significantly higher in cases than in controls, whereas the 2-OHE1 : 16*α*-OHE1 ratio was not statistically different in cases and controls.

The 2-OHE1 and 16*α*-OHE1 were moderately correlated with each other (cohort- and age-adjusted Spearman correlation *r*=0.20 in controls and 0.19 in cases). The 2-OHE1 : 16*α*-OHE1 ratio was strongly correlated with levels of 2-OHE1 (*r*=0.85 in controls and 0.87 in cases) and negatively, but less strongly, correlated with 16*α*-OHE1 (*r*=−0.30 in controls and −0.26 in cases). Correlations were low between oestrone and 2-OHE1 (*r*=0.13 in controls and 0.19 in cases), 16*α*-OHE1 (*r*=0.15 in controls and 0.21 in cases) and their ratio (*r*=0.03 in controls and 0.10 in cases). Correlations of the oestrogen metabolites and their ratio with oestradiol were in the same direction as with oestrone but weaker (data not shown).

Table 3 reports odds ratios for the associations of the two oestrogen metabolites and their ratio, with risk of endometrial cancer. In unadjusted analyses, the risk of endometrial cancer increased significantly with increasing tertiles of 2-OHE1 and 16*α*-OHE1. The associations remained significant after adjusting for known endometrial cancer risk factors, with odds ratios in the top tertile of 2.4 (95% CI=1.3–4.6; *P*_trend_=0.007) for 2-OHE1 and 1.9 (95% CI=1.1–3.5; *P*_trend_=0.03) for 16*α*-OHE1. Although the odds ratios in the top two tertiles were somewhat elevated, no significant association was observed with the 2-OHE1 : 16*α*-OHE1 ratio in unadjusted, as well as in adjusted, analyses.

Table 4 shows odds ratios for endometrial cancer risk associated with a doubling in levels of each of the two oestrogen metabolites and of their ratio, for all subjects included in the study as well as for the subset of 101 cases and 183 controls for whom oestrone and oestradiol measurements were available. Overall results were similar in the two groups, that is significant trends of increasing risk with increasing levels of 2-OHE1 and 16*α*-OHE1 and no association with the ratio, although odds ratios for 2-OHE1 and 16*α*-OHE1 were slightly higher in the subgroup than in the whole-group analyses. The table also shows that adjusting for either oestrone or oestradiol, in addition to adjusting for known risk factors, resulted in an attenuation of the odds ratios associated with 2-OHE1 and 16*α*-OHE1, which were no longer statistically significant. On the other hand, the strong associations of oestrone and oestradiol with endometrial cancer risk remained highly significant after adjusting for the two oestrogen metabolites.

Results in analyses limited to the 142 sets whose case subject had an endometrioid-type tumour were similar to the overall results (data not shown). There was no evidence of heterogeneity by cohort in any of the analyses. There was also no evidence of effect modification by age at blood donation, age at diagnosis, lag time between blood donation and diagnosis or BMI.

## Discussion

Contrary to our hypothesis, women with a higher 2-OHE1 : 16*α*-OHE1 ratio did not have a decreased risk of endometrial cancer as compared with women with a lower ratio. Regarding absolute levels of 2-OHE1 and 16*α*-OHE1, we found that both were directly associated with risk in unadjusted analyses as well as in analyses adjusting for known risk factors of endometrial cancer. These associations, though, were no longer significant after adjusting for oestrone, which is positively associated with both risk of endometrial cancer (Lukanova *et al*, 2004; Allen *et al*, 2008) and levels of metabolites, and therefore a positive confounder. Similar results were observed when adjusting for oestradiol, a more potent oestrogen than oestrone. On the other hand, the odds ratios associated with the 2-OHE1 : 16*α*-OHE1 ratio were not materially affected by adjustment for oestrone. This was not surprising, as the ratio was not correlated with the level of oestrone, which confirmed that the ratio is an index of the relative importance of these two metabolic pathways independent of the levels of oestrone.

The findings from this first prospective epidemiological study of oestrogen metabolites and endometrial cancer are in line with results from prospective studies on breast cancer, another oestrogen-related cancer. None of the seven studies on breast cancer reported significant associations overall (Meilahn *et al*, 1998; Muti *et al*, 2000; Cauley *et al*, 2003; Wellejus *et al*, 2005; Modugno *et al*, 2006; Eliassen *et al*, 2008; Arslan *et al*, 2009). Some studies reported significant associations in subgroups, but the subgroup results were not consistent across studies (Eliassen *et al*, 2008). On the whole, prospective epidemiological data do not support the hypothesis that the 2-hydroxyestrogen pathway is protective, and the 16*α*-hydroxyestrogen pathway harmful, in hormone-dependent cancers.

Although no longer significant, odds ratios associated with 2-OHE1 remained somewhat elevated after adjusting for oestrone or oestradiol. It is possible that these elevations were due to remaining confounding by the main oestrogens affecting endometrial cancer, that is, oestrone and oestradiol. However, a significant increase in risk of breast cancer with levels of 2-OHE1 has also been reported previously, although it was limited to hormone receptor-negative tumours (Eliassen *et al*, 2008). Although there is a substantial amount of experimental data supporting the hypothesis that the 2-hydroxyestrogen pathway may be protective against oestrogen-driven cancers, not all studies agree. One study showed that uterine adenocarcinomas were induced in CD-1 mice by both 2-hydroxyestradiol and 4-hydroxyestradiol (Newbold and Liehr, 2000). Both 2- and 4-hydroxyestrogens are catecholestrogens, and it has been suggested that catecholestrogens increase risk of oestrogen-mediated cancers through direct genotoxic effects, rather than through stimulation of cell proliferation via binding to oestrogen receptors (Jefcoate *et al*, 2000; Yager, 2000; Liehr and Jones, 2001; Cavalieri *et al*, 2002). Although the evidence is stronger for 4-hydroxyestrogens than for 2-hydroxyestrogens (Gaikwad *et al*, 2008), the hypothesis that catecholestrogens, including 2-hydroxyestrogens, increase risk of endometrial cancer deserves to be evaluated. Further, oestrogen can be metabolised through other pathways. New methods are now available to measure a broad range of oestrogen metabolites (Xu *et al*, 2005, 2007), which may help assess the role of various pathways in the development of breast and endometrial cancers.

Strengths of our study include its prospective design, with blood samples collected before diagnosis, and the results of our pilot study, which showed that oestrogen metabolites had good temporal reliability in our population, indicating that a single measurement reflects reasonably well a woman's average level over several years, relative to other women. Associations with known risk factors for endometrial cancer were observed in our study. Finally, in addition to adjusting for these known risk factors, we were able to control for circulating levels of the two main oestrogens, that is, oestrone and oestradiol.

Our study also has some limitations. First, the specificity and accuracy of the EIAs assays have been recently questioned (Faupel Badger *et al*, 2010). An early study comparing the EIAs with GC–MS for urinary samples from postmenopausal women had showed high correlations between the two methods for 2-OHE1, 16*α*-OHE1 and their ratio (Spearman correlation coefficients ⩾0.70; Falk *et al*, 2000). A recent, much larger study, though, found that in postmenopausal women the mean urinary concentration of 2-OHE1 was 6-fold higher, and that of 16*α*-OHE1 12-fold higher when measured by the EIAs than when measured using liquid chromatography–tandem mass spectrometry (LC–MS/MS). Further, the Spearman correlation coefficients for the ratio based on the two methods were only 0.17 for the 2-OHE1 : 16*α*-OHE1 ratio and 0.25 for the 2-pathway : 16*α*-pathway ratio (Faupel Badger, Fuhrman *et al*, 2010). Although these correlations are weak, suggesting limited specificity of the EIA assays, it is unlikely that we would have observed an inverse association between the 2-OHE1 : 16*α*-OHE1 ratio and endometrial cancer risk if we had used LC–MS/MS rather than EIAs because, although not significant, we observed a positive association between the EIA ratio and risk of endometrial cancer and because the correlation of the ratio estimates obtained by the two methods is positive.

Another limitation of our study is that concentrations of oestrogen metabolites were higher when measured in EDTA plasma (for the NSHDS) than when measured in serum (for the NYUWHS and ORDET) samples. Several reasons may have contributed to the higher concentrations observed with EDTA-plasma samples, such as binding of EDTA with cations (e.g., calcium) which are cofactors in the stabilisation of the enzymes used in assay (e.g., alkaline phosphatase) and differences in protein composition. Because the validation study we conducted showed that serum concentrations could be accurately predicted from plasma concentrations in Swedish participants for whom EDTA-plasma and serum samples collected at the same time were available, we predicted serum values for the NSHDS participants and presented results combining the three cohorts. It should be noted that analyses conducted within each cohort separately showed similar results, suggesting that the type of samples did not affect the conclusions.

In conclusion, our results do not support the hypothesis that greater metabolism of oestrogen via the 2-OH pathway, relative to the 16*α*-OH pathway, protects against endometrial cancer. Indeed our results are more suggestive of an increase in risk, rather than a decrease, with higher levels of 2-OHE1. Assays measuring a broad panel of oestrogen metabolites, including catecholestrogens, should be used in future studies to assess the role of various oestrogen metabolic pathways in the development of oestrogen-related cancers.

## Figures and Tables

**Table 1:** Description of the cohorts

**Cohort**	**Cohort size (age at enrolment, years)**	**Recruitment (enrolment period)**	**Data collection**	**Case ascertainment methods**	**Number of cases/controls**
NSHDS Umeå, Sweden	>85 000 men and women (30–70)	General population, Västerbotten County (1985–present)	Questionnaire at enrolment; data on reproductive life and hormone use updated by case–control questionnaire	Linkage to the Swedish Cancer Registry	89/164
					
NYUWHS New York City, NY, USA	14 274 women, no current use of OC or HRT (35–65)	Breast cancer screening centre (1985–1991)	Questionnaire at enrolment; prospective follow-up questionnaires; and case–control interview for reproductive life and hormone use	Follow-up questionnaires and linkages to state tumour registries (NY, NJ, FL) and the NDI	75/143
					
ORDET Milan, Italy	10 788 women, no current use of OC or HRT and no total oophorectomy (35–69)	General population, Varese Province (1987–1992)	Interview at enrolment	Linkage to the Lombardy Cancer Registry	15/29

Abbreviations: OC=oral contraceptives; HRT=hormone replacement therapy; NDI=national death index; NSHDS=Northern Sweden Disease and Health Study; NYUWHS= New York University Women's Health Study.

**Table 2:** Description of case and control subjects

	**Cases (*n*=179)**			**Controls (*n*=336)**			
**Characteristic**	***N***	**%**	**Median (10th, 90th)**	***N***	**%**	**Median (10th, 90th)**	***P*-value**
Age at blood donation, years			59.9 (51.6, 65.4)			59.9 (51.8, 65.4)	Matched
Age at diagnosis, years			66.5 (58.9, 73.8)				
Lag time between blood donation and diagnosis			7.3 (1.9–13.0)				
							
*Race*							0.41
Caucasian	163	94		312	97		
Other	11	6		13	3		
Missing	5			11			
Height, cm			162.6 (154.0, 170.2)			161.5 (154.0, 169.0)	0.49
Weight, kg			71.3 (55.0, 94.0)			65.8 (54.4, 84.4)	<0.0001
							
*Body mass index, kg m* ^−*2*^							0.001
<25	53	34		154	50		
25-<30	57	37		101	33		
⩾30	46	29		54	17		
Missing, *n*	23			27			
							
*Age at menarche, years*							0.01
<12	33	19		33	10		
12–13	82	46		161	49		
>13	61	35		132	41		
Missing, *n*	3				10		
							
*Age at menopause, years*			51.5 (46.0, 56.0)			50.0 (45.0, 55.0)	0.004
Missing, *n*			23			43	
*Nulliparous*	29	16		58	18		0.70
Missing, *n*	0			5			
*Ever used OC*	33	19		75	23		0.05
Missing, *n*	7			13			
*Ever used HRT*	59	37		80	26		0.01
Missing, *n*	23			27			
*Ever smoked*	57	34		134	42		0.09
Missing, *n*	12			17			
*History of diabetes*	13	8		17	5		0.54
Missing, n	10			11			
2-OHE1 (pg ml^−1^)			130.2 (67.0–221.4)			113.5 (61.3, 208.3)	0.03
16*α*-OHE1 (pg ml^−1^)			335.3 (255.0, 460.2)			314.0 (225.6, 436.9)	0.02
2-OHE1 : 16*α*-OHE1 ratio			0.40 (0.19, 0.69)			0.37 (0.19, 0.70)	0.58

Abbreviations: OC=oral contraceptives; HRT=hormone replacement therapy; 16*α*-OHE1=16*α*-hydroxyestrone; 2-OHE1=2-hydroxyestrone.

**Table 3:** ORs and 95% CIs for endometrial cancer according to circulating levels of 2-OHE1, 16*α*-OHE1 and the 2-OHE1 : 16*α*-OHE1 ratio

	**Tertiles**			
**Oestrogen metabolites**	**1**	**2**	**3**	***P*_trend_**
*2-OHE1*				
Cases/controls	45/125	61/112	73/99	
Crude OR (95% CI)	1.00 (reference)	1.55 (0.89, 2.70)	2.24 (1.28, 3.91)	0.005
Multivariate OR (95% CI)^a^	1.00 (reference)	1.87 (0.98, 3.55)	2.41 (1.27, 4.58)	0.007
				
*16α-OHE1*				
Cases/controls	44/127	66/107	69/102	
Crude OR (95% CI)	1.00 (reference)	1.71 (1.01, 2.91)	2.01 (1.21, 3.35)	0.007
Multivariate OR (95% CI)^a^	1.00 (reference)	2.06 (1.14, 3.70)	1.89 (1.05, 3.41)	0.03
				
*2-OHE1 : 16α-OHE1 ratio*				
Cases/controls	49/121	69/104	61/111	
Crude OR (95% CI)	1.00 (reference)	1.56 (0.92, 2.66)	1.34 (0.78, 2.31)	0.28
Multivariate OR (95% CI)^a^	1.00 (reference)	1.74 (0.95, 3.20)	1.33 (0.73, 2.44)	0.35

Abbreviations: BMI=body mass index; CI=confidence interval; OR=odds ratio; 16*α*-OHE1=16*α*-hydroxyestrone; 2-OHE1=2-hydroxyestrone.

a Adjusted for age at menarche (<12, 12, 13, >13 years and missing), age at menopause (<50, 50-52, >52 years and missing), parity (ever, never and missing), oral contraceptive use (ever, never and missing), hormone replacement therapy use (ever, never and missing), smoking (ever, never and missing) and BMI (<25, 25–30, ⩾30 kg m^−2^ and missing).

**Table 4:** Odds ratios associated with a doubling in levels of circulating oestrogens, oestrogen metabolites and 2-OHE1 : 16*α*-OHE1 ratio

**Model**	**Oestrone**	**Oestradiol**	**2-OHE1**	**16*α*-OHE1**	**2-OHE1 : 16*α*-OHE1 ratio**
*All subjects*					
Adjusted for known risk factors^a^					
OR (95% CI)			1.39 (1.01, 1.90)	1.73 (1.05, 2.85)	1.05 (0.50, 2.20)
*P*-value			0.04	0.03	0.90
					
*Subjects with oestrogen measurements*					
Adjusted for known risk factors^a^					
OR (95% CI)	2.25 (1.36, 3.71)	2.51 (1.57, 4.02)	1.76 (1.07, 2.92)	1.92 (0.93, 3.96)	1.07 (0.41, 2.81)
*P*-value	0.002	0.0001	0.03	0.08	0.89
					
Adjusted for known risk factors, 2-OHE1 and 16α-OHE1^a^					
OR (95% CI)	1.98 (1.18, 3.32)	2.26 (1.40, 3.66)			
*P*-value	0.009	0.001			
					
Adjusted for known risk factors and oestrone^a^					
OR (95% CI)			1.51 (0.89, 2.55)	1.58 (0.80, 3.15)	1.00 (0.37, 2.73)
*P*-value			0.13	0.19	0.99
					
Adjusted for known risk factors and oestradiol^a^					
OR (95% CI)			1.51 (0.89, 2.57)	1.59 (0.79, 3.22)	1.04 (0.37, 2.91)
*P*-value			0.12	0.19	0.94

Abbreviations: BMI=body mass index; CI=confidence interval; OR=odds ratio; 16*α*-OHE1=16*α*-hydroxyestrone; 2-OHE1=2-hydroxyestrone.

a Age at menarche (<12, 12, 13, >13 years and missing), age at menopause (<50, 50–52, >52 years and missing), parity (ever, never and missing), oral contraceptive use (ever, never and missing), hormone replacement therapy use (ever, never and missing), smoking (ever, never and missing), and BMI (<25, 25–30, ⩾30 kg m^−2^ and missing).
